# Serum angiopoietin-2: a promising biomarker for early diabetic kidney disease in children and adolescents with type 1 diabetes

**DOI:** 10.1007/s00431-024-05637-w

**Published:** 2024-06-17

**Authors:** Nanees Abdel-Badie Salem, Wafaa M. Ismail, Shimaa R. Hendawy, Ashraf M. Abdelrahman, Ahmed M. El-Refaey

**Affiliations:** 1 Pediatric Endocrinology and Diabetes Unit, Department of Pediatrics, Faculty of Medicine, Mansoura University, Mansoura, Egypt; 2 Mansoura University Children’s Hospital, Mansoura, Egypt; 3 Department of Clinical Pathology, Faculty of Medicine, Mansoura University, Mansoura, Egypt; 4 Department of Diagnostic Radiology, Mansoura University Children’s Hospital, Mansoura, Egypt; 5 Nephrology Unit, Department of Pediatrics, Faculty of Medicine, Mansoura University, Mansoura, Egypt

**Keywords:** Diabetic kidney disease, Children, Type 1 diabetes mellitus, Serum angiopoietin-2

## Abstract

Albuminuria has been considered the golden standard biomarker for diabetic kidney disease (DKD), but appears once significant kidney damage has already occurred. Angiopoietin-2 (Angpt-2) has been implicated in the development and progression of DKD in adults. We aimed to explore the association of serum Angpt-2 levels with DKD in children and adolescents with type 1 diabetes mellitus (T1DM) of short duration (3–5 years) and to evaluate the predictive power of serum Angpt-2 in the early detection of DKD prior to the microalbuminuric phase. The current cross-sectional study included 90 children divided into three age and sex-matched groups based on urinary albumin-to-creatinine ratio (UACR): microalbuminuric diabetic group (*n* = 30), non-albuminuric diabetic group (*n* = 30), and control group (*n* = 30). All participants were subjected to anthropometric measurements, serum Angpt-2 and fasting lipid profile (total cholesterol, triglycerides, LDL-C, HDL-C, and Non-HDL-C) assessment. Glomerular filtration rate was estimated based on serum creatinine (eGFR-Cr). Higher serum Angpt-2 levels were detected in both diabetic groups compared to controls and in microalbuminuric compared to non-albuminuric diabetic group. There was no detected significant difference in eGFR-Cr values across the study groups. Serum Angpt-2 was positively correlated with triglycerides, LDL, Non-HDL-C, HbA1c, and UACR, while UACR, HbA1c, and Non-HDL-C were independent predictors for serum Angpt-2. Serum Angpt-2 at level of 137.4 ng/L could discriminate between microalbuminuric and non-albuminuric diabetic groups with AUC = 0.960 and at level of 115.95 ng/L could discriminate between the non-albuminuric diabetic group and controls with AUC = 0.976.

*Conclusion*: Serum Angpt-2 is a promising potent biomarker for the detection of early stage of DKD in childhood T1DM before albuminuria emerges.

**Table:** 

**What is Known?** • *Urine albumin-to-creatinine ratio (UACR) and glomerular filtration rate (GFR) are the golden standard but late biomarkers for DKD.* • *Angiopoietin-2 has been implicated in the development and progression of DKD in adults with diabetes, but has not been explored in T1DM children with DKD.*
**What is New?** • *Higher serum angiopoietin-2 was detected in diabetic groups compared to controls and in microalbuminuric compared to non-albuminuric group.* • *Angiopoietin-2 correlated positively with triglycerides, LDL, Non-HDL-C, HbA1c, and UACR.* • *Serum angiopoietin-2 is a promising early diagnostic biomarker for DKD in children with T1DM.*

**What is Known?**

• *Urine albumin-to-creatinine ratio (UACR) and glomerular filtration rate (GFR) are the golden standard but late biomarkers for DKD.*

• *Angiopoietin-2 has been implicated in the development and progression of DKD in adults with diabetes, but has not been explored in T1DM children with DKD.*

**What is New?**

• *Higher serum angiopoietin-2 was detected in diabetic groups compared to controls and in microalbuminuric compared to non-albuminuric group.*

• *Angiopoietin-2 correlated positively with triglycerides, LDL, Non-HDL-C, HbA1c, and UACR.*

• *Serum angiopoietin-2 is a promising early diagnostic biomarker for DKD in children with T1DM.*

## Introduction

Diabetic kidney disease (DKD) is a common serious microvascular complication in diabetic patients and is considered a major contributor to morbidity and premature mortality in young adults with childhood-onset type 1 diabetes mellitus (T1DM) [1, 2].

In diabetic patients, renal functional deterioration is the result of specific heterogeneous renal structural changes, namely, thickening of the glomerular basement membrane and mesangial expansion, appearing soon after DM onset (1.5–5 years), but DKD remains in a clinically silent phase for a long period [3]. Clinical and biological abnormalities (micro/macroalbuminuria) and progressive decline in glomerular filtration rate (GFR) will develop over 10–25 years [4–7].

According to the recent International Society of Pediatric and Adolescent Diabetes guidelines (ISPAD, 2022), screening for DKD in children with T1DM by albuminuria and estimated GFR (eGFR) should start at puberty or from age 11 years whichever is earlier, with 2–5 years of diabetes duration, and repeated annually thereafter [8].

Kidney Disease Improving Global Outcomes and Diabetes Work Groups (KDIGO 2020) defined persistent albuminuria (formerly microalbuminuria) as a urine albumin-to-creatinine ratio (UACR) of 30–299 mg/g creatinine in at least 2 of 3 urine samples over a 3–6-month period [9]. Once albuminuria develops, urine albumin excretion continues to rise and progress to overt proteinuria, formerly termed “macroalbuminuria,” defined as an UACR value of ≥ 300 mg/g creatinine, heralds the onset of overt DKD and is thought to inexorably lead to impaired GFR (defined as an eGFR < 60 mL/min/1.73 m^2^) and eventually end-stage renal disease [9]. Persistent albuminuria has classically been considered the earliest sign of DKD and occurs in 26% of children and adolescents after 10 years and in 51% after 19 years of diabetes [6]. It reflects widespread endothelial dysfunction and indicates microvascular damage and corresponds to diabetic nephropathy stage 3. Thus, microalbuminuria appears once significant kidney damage has already occurred [10].

Considering that prediction and recognition of DKD at early stage before microalbuminuria occurrence have a pivotal role in providing timely management to prevent or delay kidney damage [11, 12], it is crucial to identify earlier, more specific and sensitive biomarkers with higher predictability for DKD alternative to albuminuria-based method.

Many urinary and serum biomarkers involved in the pathogenic mechanisms of DKD have been evaluated for their possible role in the detection of early DKD in adults with type 2 diabetes [13, 14]. However, the current tools for early diagnosis of DKD in children and adolescents are few and flawed [15]. None of these putative markers is currently a part of routine clinical care in children or adults with DKD though.

In general, the pathogenesis of DKD involves a multifactorial interaction of metabolic and hemodynamic factors. Recently, endothelial dysfunction/angiogenesis was regarded as one of the earliest mechanisms involved in the pathophysiology and progression of diabetes-related macro-and microangiopathies. Factors contributing to development of endothelial dysfunction are derangements in vascular tone, glucotoxicity, and dysbalance in production of vascular growth factors regulating the physiology of the vascular wall [16].

Angiopoietins are essential vascular growth factors that tightly control angiogenesis, vascular permeability, inflammation, and remodeling, which also play an important role in the glomerular capillaries’ homeostasis in both physiology and disease [17, 18]. Two major isoforms regulate vascular homeostasis, namely, angiopoietin-1 (Angpt-1) and angiopoietin-2 (Angpt-2), behaving antagonistically to each other to sustain vascular endothelium homeostasis. While Angpt-1 typically acts as the endothelium-protective mediator, Angpt-2 inhibits binding of Angpt-1 to the endothelium-specific tyrosine kinase-2 (Tie-2) receptor and promotes endothelium permeability and vascular destabilization. Furthermore, Angpt-2 stimulates endothelial cell proliferation as well as promotes neovascularization in synchronous action with vascular endothelial growth factor-A [18].

Increasing evidence suggests that the upregulation of Angpt-2 is harmful for kidney physiology and function. Overexpression of Angpt-2 and abnormal alterations in Angpt-1/Angpt-2 ratio cause excess angiogenesis and inflammation that destabilize glomerular endothelial cells and affect podocytes in the paracrine fashion, inducing the decay of glomerular filtration barrier function [19].

Despite growing evidence for the crucial role of angiopoietins in the development and progression of DKD in adults [20, 21], there are scarce data on angiopoietins in children with T1DM [22] and whether serum angiopoietins can predict early DKD in children with T1DM has not been explored. The current study aimed to explore the association of serum Angpt-2 levels with DKD in children with T1DM of short duration (3–5 years) and to evaluate the predictive power of serum Angpt-2 in the early detection of DKD prior to the microalbuminuric phase.

## Subjects and methods

This case–control study was conducted between June 2022 and June 2023 and enrolled 60 children and adolescents with stable T1DM who were maintained on intensive insulin therapy and a control group of 30 age and sex‐matched healthy children. Based on ISPAD guidelines 2022 [8], we selected children at puberty or from age 11 years whichever is earlier, with 2–5 years of diabetes duration. Children with T1DM were enrolled sequentially from the outpatient clinic of Pediatric Diabetes at Mansoura University Children Hospital, Mansoura, Egypt. Patients with associated congenital or acquired kidney disease, thyroid dysfunction, history of recent infection, or recent diabetic ketoacidosis and those who received nephrotoxic drugs or glucocorticoids were excluded.

The study protocol was approved by the local ethics committee of Faculty of Medicine, Mansoura University-Institutional Research Board (Reference no. MS.21.01.1358). Parents of enrolled children provided informed consent before participation in the study.

### Sample size estimation

The sample size was estimated using an online sample size calculator program (G*Power 3) based on the results of study published by Aly et al. [23] that gave a serum Angpt-2 level 155.6 ± 11.8 pg/mL to the microalbuminuric group and a serum Angpt-2 level 131 ± 20.7 pg/mL to the non-albuminuric group. The calculated sample size of the study will be 21 participants for each group, assuming 95% confidence interval, 5% level of significance, 10% margin of error, and 85% power of the study. The sample size was increased to 30 participants for each group to increase the study power.

### Method

Children with T1DM attending Mansoura University Children Hospital who fulfilled inclusion criteria underwent full medical history, standard physical examination, and biochemical evaluation during their routine follow-up visits at Pediatric Diabetes outpatient clinic. The following clinical variables were collected: age, sex, age at onset of diabetes, duration of diabetes, and total daily insulin dose (IU/kg/day). The results of HbA1c (%) over the last year were extracted from the medical files of children with T1DM, and then, average HbA1c was obtained.

Weight and height were evaluated by standard methods. Body mass index (BMI) was calculated as weight (kg)/height (m^2^), and then, BMI SD score (z-score) for age and sex was calculated based on reference data for healthy Egyptian children [24]. Pubertal development was determined in all study participants using Tanner classifications of breast development in females and genital development in males [25].

Blood pressure was evaluated using the conventional mercury manometer using standard technique; the results then were plotted on blood pressure curves according to the age, sex, and height centiles. Hypertension is diagnosed if the average systolic blood pressure (SBP) and/or diastolic blood pressure (DBP) is > 95th percentile for age, gender, and height on more than three measures [26].

### Laboratory investigations

Three-milliliter venous samples were aseptically collected early morning after subjects had fasted for at least 8 h and centrifuged at 4000 rpm for 10 min, and the sera were stored at − 80 °C until being assessed for serum creatinine, lipids, and serum Angpt-2 levels.

Serum creatinine (Cr, mg/dL) was measured on a Dimension Xpand Plus Chemistry Analyzer using its kits supplied by Siemens Technology (USA). Fasting lipid profile was determined including serum total cholesterol (TC) and triglycerides (TGs) measured by a colorimetric kit supplied by Spinreact (Girona, Spain), and serum high-density lipoprotein cholesterol (HDL-C) was measured by a colorimetric kit supplied by Human Diagnostics (Wiesbaden, Germany). Low-density lipoprotein cholesterol (LDL-C) was estimated using the Friedewald formula: LDL (mg/dL) = [TC − HDL] − TGs/5. Non-HDL-C was calculated by subtracting HDL-C from TC and reflects the cholesterol in all atherogenic lipoprotein particles. Serum Angpt-2 level (ng/L) was assessed using the human Angpt-2 sandwich enzyme-linked immunosorbent (ELISA) kit (supplied by BT LAB bioassay technology laboratory, Cat. No. E1221Hu, China).

Estimated glomerular filtration rate (eGFR; mL/min/1.73 m^2^) was calculated based on serum creatinine (Cr) values using updated Schwartz formula: serum creatinine-based eGFR (eGFR-Cr) = K*height (cm)/serum Cr (mg/dL), where *K* is 61.9 in males aged 13 years and older and 48.6 in other children [27].

Complete urine analysis and culture were performed to exclude urinary tract infection in all subjects. Five milliliter of fresh voided first morning mid-stream urine has been collected in a sterile container and stored at − 80 °C till analysis. Urinary creatinine (mg/dL) was measured on Dimension Expand Plus (Siemens Diagnostic, USA) using its commercial kits. Urine microalbumin (mg/dL) was determined using ELISA kit supplied by ORGENTEC Diagnostika (Mainz, Germany). In order to eradicate the effect of urine dilution or concentration on the urinary markers, the results were described as the ratio of urinary microalbumin/creatinine ratio (UACR) expressed in mg/g creatinine. Based on the results of UACR, children with T1DM were subdivided into two groups; non-albuminuric group (*n* = 30; UACR value < 30 mg/g creatinine) and microalbuminuric group (*n* = 30; UACR value 30–299 mg/g creatinine) based on the results of at least 2 of 3 urine samples over a 3–6-month period.

HbA1c was measured by quantitative colorimetric ion exchange resin chromatography kits provided by Stan Bio Laboratory (Boerne, TX, USA) (procedure no. 0350). Calibrators referenced to National Glycohemoglobin Standardization Program and values of HbA1c were presented as the unit of a percentage (%).

### Statistical analysis

All statistical analyses were performed using IBM SPSS software package version 20.0. Qualitative variables were presented as number and percent [*n* (%)] and compared by chi-square test. Quantitative variables were tested for normality using the Kolmogorov–Smirnov test. Normally distributed variables were expressed as mean and standard deviation (mean ± SD), and non-normally distributed variables were presented as median and interquartile range (IQR). Quantitative data for two groups were compared by independent-samples *t*-test or its non-parametric equivalent, Mann–Whitney *U* test. Quantitative data for the three groups were compared by one-way analysis of variance (ANOVA) test or its non-parametric equivalent, Kruskal–Wallis test. Spearman correlation was used to determine the strength and direction of a linear relationship between two continuous non-normally distributed variables, and Pearson correlation was used for parametric correlation. Linear regression analysis was performed to identify the predictors of serum Angpt-2 level. Receiver operator characteristic (ROC) curve was constructed to evaluate the diagnostic value of serum Angpt-2 as a biomarker in the prediction of microalbuminuria in children with T1DM. Area under the curve (AUC), specificity, and sensitivity were computed based on the ROC. The best cutoff point with relevant sensitivity and specificity was defined. Results were considered statistically significant for any test if *P* < 0.05.

## Results

In the current study, three groups were included: microalbuminuric diabetic group (*n* = 30; 16 male), non-albuminuric diabetic group (*n* = 30; 18 male), and control group (*n* = 30; 17 male).

The clinical and biochemical characteristics of the study groups are presented in Table 1. The three groups were matched for age, sex, and pubertal status (*P* > 0.05) with higher frequency of pubertal vs. pre-pubertal children in both non-albuminuric and microalbuminuric groups; thus, we studied each group as a whole without being subclassified based on pubertal status.

**Table 1:** Clinical and biochemical characteristics of the study groups

	Control (*n* = 30)	Non-albuminuric (*n* = 30)	Microalbuminuric (*n* = 30)	*P*
Age (year)	12.5 ± 2.0	13.2 ± 1.78	12.9 ± 1.85	0.355
Sex				
Male *n* (%)	17 (56.7)	18 (60)	16 (53.3)	0.654
Female *n* (%)	13 (43.3)	12 (40)	14 (46.7)	
Pubertal status				
Pre-pubertal *n* (%)	15 (50)	14 (46.7)	12 (40)	0.353
Pubertal *n* (%)	15 (50)	16 (53.3)	18 (60)	
Age at DM onset (year)	–	8.5 (6.0–10.25)	7.0 (4.0–10.25)	0.593
Insulin TDD (IU/kg/d)	–	0.9 (0.8–1.2)	1.1 (0.75–1.6)	0.123
BMI z-score	0.85 (− 1.2–1.10)	0.61 (− 1.01–1.21)	0.76 (− 0.98–1.50)	0.745
SBP (mmHg)	105.7 ± 10.5	110.2 ± 8.34	107.8 ± 10.18	0.206
HbA1c (%)	4.37 (4.0–5.3)	8.05 (7.5–8.7)^†^	9.3 (8.5–10.6)^†,‡^	< 0.001*
Total cholesterol (mg/dL)	156 (150–162)	164 (157.7–171)^†^	190 (196.2–210)^†,‡^	< 0.001*
Triglycerides (mg/dL)	68.4 (63–87.5)	72.8 (70–98.4)	76.5 (60–112.5)	0.254
LDL-C (mg/dL)	85.9 (76.5–100)	91.5 (79.2–120.7)	120.1 (104.5–139)^†,‡^	< 0.001*
HDL-C (mg/dL)	60.0 (47.0–66.5)	57.3 (48.0–72.5)	49.0 (45.0–60.2)^†,‡^	0.039*
Non-HDL-C (mg/dL)	101.5 (92–101.5)	108.5 (93–121)	135.5 (120.5–145)^†,‡^	< 0.001*
Serum creatinine (mg/dL)	0.65 ± 0.13	0.71 ± 0.11	0.67 ± 0.12	0.151
eGFR-Cr (mL/min/1.73 m^2^)	110 (108.7–116)	107.5 (80–115.2)	100.7 (71.0–118.2)	0.208
UACR (mg/g creatinine)	9.8 (8.3–10.9)	11.9 (9.3–16.6)	72.86 (51.9–116.9)^†,‡^	< 0.001*
Serum angiopoietin-2 (ng/L)	90.5 (73.3–101.8)	125.3 (99.8–143.5)^†^	148 (109.5–201.3)^†,‡^	< 0.001*

Data presented as mean ± SD, median (IQR), or frequency number (%)

*BMI* body mass index, *Cr* creatinine, *eGFR-Cr* creatinine-based estimated glomerular filtration rate, *HDL-C* high-density lipoprotein cholesterol, *LDL-C* low-density lipoprotein cholesterol, *SBP* systolic blood pressure, *TDD* total daily dose, *UACR* urinary albumin-to-creatinine ratio

^*^Statistically significant difference (*P* < 0.05)

^†^Significant difference with the control group

^‡^Significant difference with the non-albuminuric group

No significant differences were detected between the two diabetic groups as regards diabetes-related variables including age at T1DM onset and total daily insulin dose (IU/kg/day) (*P* > 0.05). The median duration of T1DM in both groups was 3.5 years (3–5 years).

The included subjects showed normal blood pressure and serum creatinine. The eGFR-Cr values of all participants were within the normal reference range for age and sex based on National Kidney Foundation’s Kidney Disease Outcomes Quality Initiative (KDOQI) clinical practice guidelines for chronic kidney disease in children and adolescents [28]. No significant differences were detected in BMI z-score, SBP, DBP, serum creatinine, and eGFR-Cr across the three studied groups (*P* > 0.05).

Significantly higher TC, LDL-C, and Non-HDL-C and lower HDL-C levels were detected in microalbuminuric compared to non-albuminuric and control groups, and only significantly higher TC was detected in non-albuminuric compared to control group. Significantly higher HbA1c, UACR, and serum Angpt-2 values were detected in microalbuminuric and non-albuminuric diabetic groups compared to the control group and in the microalbuminuric group compared to the non-albuminuric group (Table 1).

Correlation analysis among children with T1DM revealed that serum Angpt-2 level was positively correlated with TGs (*P* = 0.006), LDL-C (*P* = 0.004), Non-HDL-C (*P* = 0.034), HbA1c (*P* = 0.001), and UACR (*P* < 0.001). Otherwise, no significant correlations could be detected with other parameters (Table 2).

**Table 2:** Correlation analysis between serum angiopoietin-2 level (ng/L) and clinical and biochemical parameters among children with T1DM

	Serum angiopoietin-2 level (ng/L)	
	*r*	*P* value
Age (years)	0.158	0.228
Age at onset of T1DM (years)	0.066	0.620
Duration of T1DM (years)	0.008	0.951
BMI z-score	− 0.191	0.144
SBP (mm/Hg)	0.025	0.850
Total cholesterol (mg/dL)	0.216	0.098
Triglycerides (mg/dL)	0.353	0.006*
LDL-C (mg/dL)	0.369	0.004*
HDL-C (mg/dL)	− 0.127	0.333
Non-HDL-C (mg/dL)	0.274	0.034*
HbA1C (%)	0.413	0.001*
eGFR-Cr (mL/min/1.73 m^2^)	− 0.153	0.242
UACR (mg/g creatinine)	0.673	< 0.001*

*r*: Spearman’s rank-order correlation

^*^Statistically significant difference (*P* < 0.05)

*BMI* body mass index, *Cr* creatinine, *eGFR-Cr* creatinine-based estimated glomerular filtration rate, *HbA1c* glycated hemoglobin, *HDL-C* high-density lipoprotein cholesterol, *LDL-C* low-density lipoprotein cholesterol, *SBP* systolic blood pressure, *UACR* urinary albumin-to-creatinine ratio

Linear regression analysis for identifying the predictors for serum Angpt-2 level revealed that UACR, HbA1c, and Non-HDL-C were the predictors for serum Angpt-2 level (*P* < 0.001, *P* = 0.012, and *P* = 0.001, respectively) (Table 3).

**Table 3:** Linear regression analysis for the predictors of serum angiopoietin-2 level

	Unstandardized coefficient		Standardized coefficient	*T*	*P*
	*B*	Standard error	Beta		
UACR	0.131	0.022	0.565	6.071	< 0.001*
HbA1c	2.659	1.024	0.239	2.596	0.012*
Non-HDL-C	0.167	0.049	0.306	3.392	0.001*

^*^Significant *P* < 0.05

*HbA1c* glycated hemoglobin, *HDL-C* non-high-density lipoprotein cholesterol, *UACR* urine albumin-to-creatinine ratio

**Fig. 1:**
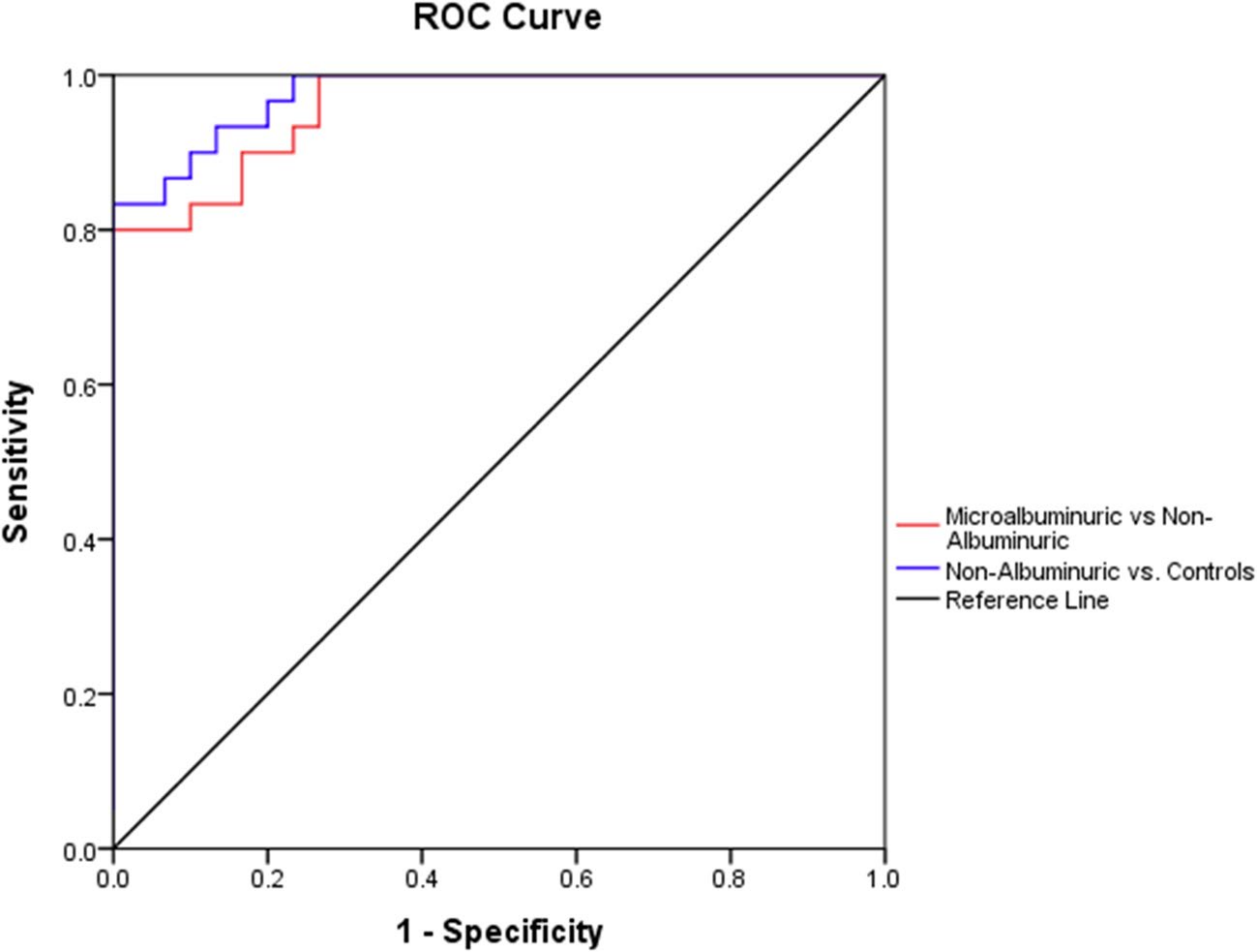
ROC curves of the diagnostic accuracy of serum Angpt-2 in prediction of DKD in children with type 1 diabetes

The diagnostic accuracy of serum Angpt-2 in prediction of DKD among children with T1DM was analyzed with construction of ROC curve. The optimal cutoff value for serum Angpt-2 to discriminate between microalbuminuric and non-albuminuric diabetic groups was at 137.4 ng/L with AUC = 0.960 with 80% sensitivity and 96.7% specificity, while the optimal cutoff value for serum Angp-2 to discriminate between the non-albuminuric diabetic group and the control group was at 115.95 ng/L with AUC = 0.976 with 86.7% sensitivity and 93.3% specificity (*P* < 0.001) (Table 4 and Fig. 1).

**Table 4:** Diagnostic accuracy of serum Angpt-2 in prediction of DKD in children with type 1 diabetes

	AUC	*P value*	95% CI	Cutoff value	Sensitivity (%)	Specificity (%)
Non-albuminuric vs. microalbuminuric diabetic groups	0.960	< 0.001*	0.919–1.001	137.4	80	96.7
Non-albuminuric diabetic group vs. control group	0.976	< 0.001*	0.946–1.005	115.95	86.7%	93.3%

*AUC* area under the curve, *CI* confidence interval

## Discussion

Considering the heavy impact of childhood diabetes on morbidity and mortality later in life, it is time to refocus efforts and resources for identification of novel diagnostic biomarkers of early silent stages of DKD in this highly vulnerable population that would facilitate the development of more effective monitoring, prevention, and treatment modalities.

In the current study, we explored the diagnostic value of serum Angpt-2 as an early biomarker for DKD in children with T1DM of short duration (3–5 years) before microalbuminuria emerges which appears once significant kidney damage has already occurred and corresponds to DKD stage 3 [10].

The study included two diabetic groups, microalbuminuric and non-albuminuric, who were matched for age, sex, and pubertal status. No significant differences were detected between the two groups as regards age at onset and duration of T1DM, blood pressure, and BMI-Z. Moreover, no significant differences in eGFR-Cr across the study groups were observed. This finding indicating that conventional creatinine-based eGFR method was unable to reflect early renal affection among children with T1DM in the current study. This finding is consistent with the results of prior studies [29–33], supporting the evidence that GFR decline develops over a longer period (10 years to 25 years) after the onset of diabetes [4].

The results of the current study revealed significantly higher serum Angpt-2 values in the microalbuminuric group compared to both non-albuminuric and control groups and in the non-albuminuric group compared to the controls. In addition, serum Angpt-2 level was found to be positively correlated with TGs, LDL-C, Non-HDL-C, HbA1c, and UACR. Among the previous biochemical parameters, Non-HDL-C, HbA1c, and UACR were the predictors for serum Angpt-2 in children with T1DM.

On reviewing the literature, only few studies reported data on Angpt-2 in adults with T1DM [33, 34] and scarce data on Angpt-2 in children with T1DM [22]. However, whether serum Angpt-2 can predict early DKD in children with T1DM has not been explored.

Consistent with our finding, El-Asrar with colleagues reported significantly higher serum Angpt-2 levels in a group of children with T1DM with and without microvascular complications including nephropathy, neuropathy, and retinopathy compared with controls. In addition, serum Angpt-2 was higher in children with microalbuminuria than normoalbuminuric group and positive associations were observed between serum Angpt-2 level and each of fasting blood glucose, HbA1c, serum creatinine, high-sensitivity C-reactive protein (hs-CRP), and with carotid and aortic intima-media thickness [22]. The authors suggested that elevated Angpt-2 levels in children with T1DM reflect inflammation and vascular dysfunction.

In adult patients with T1DM and DKD, Sokolovska and colleagues demonstrated increased concentration of serum Angpt-2 and reported associations between serum Angpt-2 and eGFR and albuminuria that remained significant after adjustment for covariates (age, sex, diabetes duration, arterial hypertension, BMI, smoking, HbA1C, and serum lipids). Therefore, the authors concluded that Angpt-2 is an independent predictor of kidney function and DKD [33]. In a longitudinal study conducted by Khairoun et al., elevated Angpt-2/Angpt-1 ratio was observed in adult patients with T1DM and DKD. Interestingly, the ratio was normalized 1 year after simultaneous kidney-pancreas transplantation [34].

Contrary to the scarce data concerned with Angpt-2 in T1DM in children [22] and in adults [33, 34], Angpt-2 has been extensively studied in adults with T2DM with comparable results. In these studies, serum Angpt-2 levels were higher in patients with T2DM compared to healthy individuals and in diabetic patients with poor glycemic control and those with chronic diabetes-related vascular complications [23, 35–43]. Therefore, Angpt-2 has been implicated in the development and progression of DKD in adults with T2DM [20, 21].

It has been postulated that chronic hyperglycemia leads to the accumulation of advanced glycation end products and mitochondrial overproduction of reactive oxygen species that causes the upregulation of Angpt-2 mRNA, which has been reported to promote vascular permeability, destabilization, and sprouting, further inducing microvascular and macrovascular complications [44, 45].

Furthermore, in a large study in Taiwan, Tsai and colleagues found a stepwise increase in serum Angpt-2 levels with urine PCR and reported association of Angpt-2 levels with renal deterioration estimated by eGFR and also demonstrated that Angpt-2 can independently predict adverse clinical outcomes, including commencing dialysis, rapid renal function decline, major adverse cardiovascular events, or all-cause mortality in DKD [46]. Unfortunately, there is no indication on type of diabetes in this study.

Moreover, urinary Angtp-2 level in DKD was explored by many studies. He with colleagues reported that tumor necrosis factor-alpha and 8-hydroxy-2′-deoxyguanosine were associated with elevated urinary Angpt-2 level in adult patients with T2DM and albuminuria [47].

In a study conducted by Chen et al., urinary Angpt-2 levels were increased in a stepwise manner in T2DM patients with various degrees of kidney damage in particular normoalbuminuric group and suggested that Angpt-2 may be an earlier measurable indicator of tubular impairment before the onset of clinical symptoms or signs, such as microalbuminuria [43].

Regarding the associations of Angpt-2 with clinical and biochemical variables in T2DM, Rasul et al. reported significant correlation between levels of Angpt-2 and HOMA-IR index, eGFR, and BMI, but not HbA1C [37]. In contrast, Lim with colleagues reported HbA1C as a predictor of Angpt-2 concentration and observed improvement in Angpt-2 levels in patients with T2DM after multifactorial intervention [35]. In addition, Angpt-2 levels are associated with indexes of endothelial damage/dysfunction in T2DM [35, 39]. Martynov et al. reported a relation between increased Angpt-2 concentration and increased albuminuria as well as reduction of GFR in adults with T2DM [38]. In the study conducted by Chen et al., no significant correlations were found between serum or urinary Angpt-2 and age, HbA1c, blood pressure, in addition to lipid profile [43]. Aly et al. found plasma Angpt-2 levels steadily increased with the progression of albuminuria and renal impairment and identified significant positive correlation of plasma Angpt-2 level with mean arterial pressure, CRP, and HbA1c and negatively correlated with eGFR, but no detected correlations between plasma levels of Angpt-2 with microalbuminuria and UACR [23]. The inconsistency between our findings and previous studies regarding Angpt-2 associations can be attributed to differences in the age of diabetic patients and the type and duration of diabetes; also, diabetic adult patients in previous studies were at advanced stages of DKD, mostly hypertensive or had associated other diabetes-related microvascular or macrovascular complications.

In the current study, we explored for the first time the predictive power of serum Angpt-2 as a biomarker for DKD in children with T1DM through construction of ROC curve. Interestingly, serum Angpt-2 demonstrated excellent diagnostic accuracy in discrimination between the studied groups. The optimal cutoff value for serum Angpt-2 to discriminate between microalbuminuric and non-albuminuric diabetic groups was at 137.4 ng/L with AUC = 0.960, 80% sensitivity, and 96.7% specificity, while the optimal cutoff value for serum Angpt-2 to discriminate between the non-albuminuric diabetic group and the control group was at 115.95 ng/L with AUC = 0.976, 86.7% sensitivity, and 93.3% specificity. This observation highlights the potential role of serum Angpt-2 as promising biomarker in the early detection of DKD prior to the microalbuminuric phase.

In agreement with our results, Aly et al. explored the validity of plasma Angpt-2 as a predictor of different stages of DKD among adult patients with T2DM who were classified into five groups: non-albuminuric, microalbuminuria, macroalbuminuria, macroalbuminuria complicated to renal impairment, and ESRD on top of DKD. The authors demonstrated that plasma Angpt-2 levels have high sensitivity and specificity in prediction of DKD at different cutoff values among studied groups [23].

Taking the results of the current study and all previous studies together, it is quite evident that serum Angpt-2 can serve as a useful diagnostic and prognostic marker for DKD in clinical settings, guiding the physicians to make proper early interference in the near future. Interestingly, manipulation of local and systemic angiopoietins could represent an attractive therapeutic target for patients with diabetic microvascular complications [48, 49]. Early studies in patients with diabetes with macular edema have shown that administration of AKB-9778 (a vascular endothelial protein tyrosine phosphatase that promotes Tie-2 receptor activation) for 4 weeks reduced macular edema and improved vision, without demonstrating any safety concerns [50]. Future studies might address the role of Tie-2 activation in diabetic glomerular disease.

## Study limitations and strengths

Notably, some limitations should be acknowledged when interpreting the findings of the current study. The cross-sectional design of the current study precluded the identification of the causal direction between serum Angpt-2 and DKD in children with T1DM and the small sample size that was related to the children being recruited from a single-center and the strict exclusion criteria also precluded the reliable evaluation of serum Angpt-2 at different stages of DKD. Nevertheless, our study is the first to explore the association of serum Angpt-2 levels with DKD in children and adolescents with T1DM and to evaluate the predictive power of serum Angpt-2 in the early detection of DKD prior to the microalbuminuric phase, thus providing relevant data for future researches.

## Conclusion

The results of the current study highlight the promising role of serum Angpt-2 as a diagnostic biomarker in the early silent stage of DKD prior to the microalbuminuric phase and support previously reported findings in adults with T1DM and T2DM, suggesting a potential role of Angpt-2 in the pathogenesis of DKD and raising its potentials for clinical and therapeutic applications in diabetes-related angiopathy especially in DKD in the near future.

Future longitudinal prospective studies with large sample size are essential to evaluate the predictive power of serum Angpt-2 for DKD to identify children at high risk of diabetic complications especially DKD before any structural damage occurs and to identify the exact role of serum Angpt-2 in monitoring the severity and progression of DKD, as well as to investigate the potential benefits of drugs targeting Angpt-2, Angpt-2 inhibitors, as a novel promising strategy to alleviate the development of microvascular damage and endothelial dysfunction in children with DKD.

## Data Availability

No datasets were generated or analyzed during the current study.

## Abbreviations

Angpt: Angiopoietin
BMI: Body mass index
DBP: Diastolic blood pressure
DKD: Diabetic kidney disease
eGFR-Cr: Serum creatinine-based estimated glomerular filtration rate
ESRD: End-stage renal disease
HbA1c: Glycated hemoglobin
HDL-C: High-density lipoprotein cholesterol
ISPAD: International Society of Pediatric and Adolescent Diabetes guidelines
LDL-C: Low-density lipoprotein cholesterol
Non-HDL-C: Non-high-density lipoprotein cholesterol
SBP: Systolic blood pressure
T1DM: Type 1 diabetes mellitus
T2DM: Type 2 diabetes mellitus
TC: Total cholesterol
TGs: Triglycerides
Tie-2: Tyrosine kinase-2 receptor
UACR: Urine albumin-to-creatinine ratio
